# Development of a sandwich ELISA to detect circulating, soluble IRAP as a potential disease biomarker

**DOI:** 10.1038/s41598-023-44038-1

**Published:** 2023-11-24

**Authors:** Anika Vear, Claudia Thalmann, Kristina Youngs, Natalie Hannan, Tracey Gaspari, Siew Yeen Chai

**Affiliations:** 1 Department of Physiology, Biomedicine Discovery Institute, Monash University, Clayton, VIC 3800 Australia; 2 Department of Obstetrics and Gynaecology, University of Melbourne, Heidelberg, VIC 3084 Australia; 3 Mercy Perinatal, Mercy Hospital for Women, Heidelberg, VIC 3084 Australia; 4 Department of Pharmacology, Biomedicine Discovery Institute, Monash University, Clayton, VIC 3800 Australia

**Keywords:** Antibody generation, Antibody isolation and purification, ELISA, Proteases, Biomarkers

## Abstract

There is growing interest in the use of the enzyme, insulin regulated aminopeptidase (IRAP), as a biomarker for conditions such as cardio-metabolic diseases and ischemic stroke, with upregulation in its tissue expression in these conditions. However, quantification of circulating IRAP has been hampered by difficulties in detecting release of the truncated, soluble form of this enzyme into the blood stream. The current study aimed to develop a sandwich ELISA using novel antibodies directed towards the soluble portion of IRAP (sIRAP), to improve accuracy in detection and quantification of low levels of sIRAP in plasma. A series of novel anti-IRAP antibodies were developed and found to be highly specific for sIRAP in Western blots. A sandwich ELISA was then optimised using two distinct antibody combinations to detect sIRAP in the low nanogram range (16–500 ng/ml) with a sensitivity of 9 ng/ml and intra-assay variability < 10%. Importantly, the clinical validity of the ELISA was verified by the detection of significant increases in the levels of sIRAP throughout gestation in plasma samples from pregnant women. The specific and sensitive sandwich ELISA described in this study has the potential to advance the development of IRAP as a biomarker for certain diseases.

## Introduction

**Figure 1:**
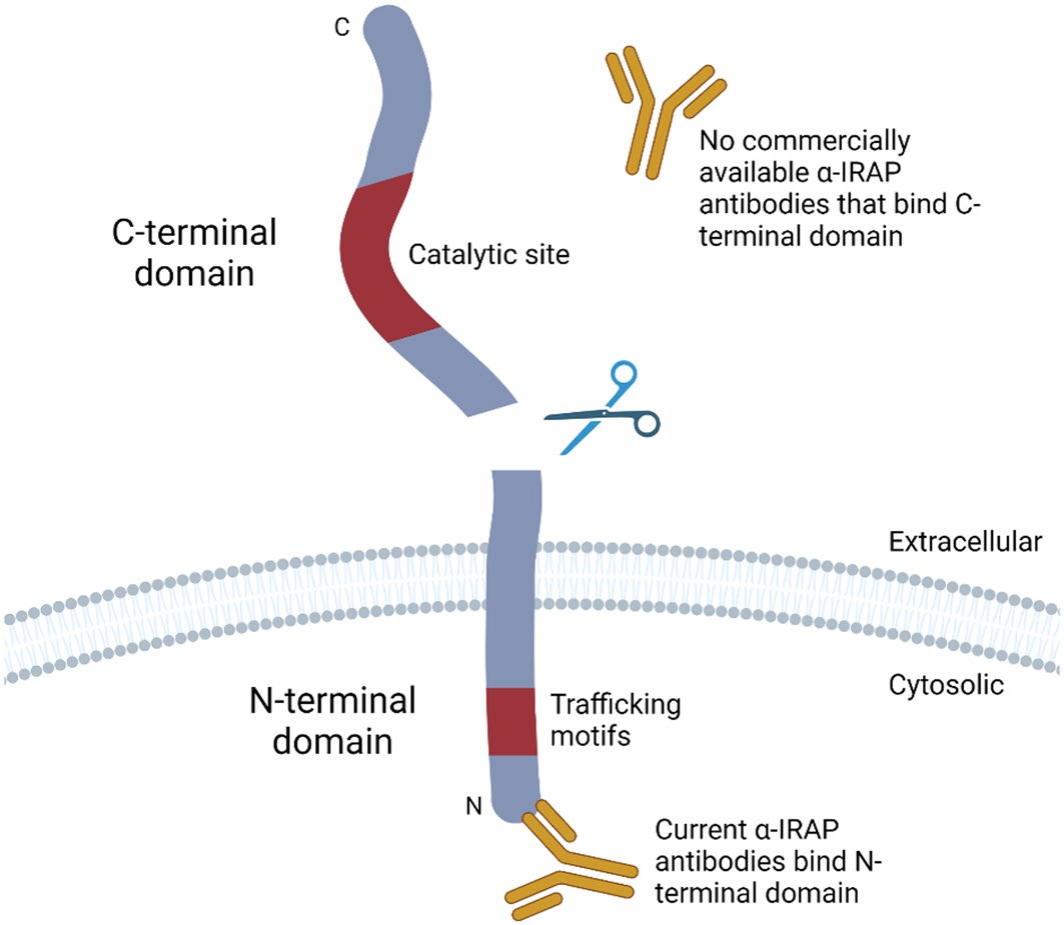
The C-terminal domain of IRAP can be secreted into the circulation/extracellular milieu. IRAP contains two functional domains: a cytosolic N-terminal domain that contains trafficking motifs and an extracellular/intra-luminal C-terminal domain that contains the catalytic site. Following cleavage at a site near the transmembrane region, the C-terminal domain can be secreted. Current anti-IRAP antibodies that are commercially available only target the N-terminal domain.

Insulin regulated aminopeptidase (IRAP) is a type II transmembrane zinc-dependent metallopeptidase which belongs to the M1 aminopeptidase family. It has two functional domains: the extracellular/intraluminal C-terminal domain which contains the catalytic site and regulates the levels of circulating peptide hormones, and the cytosolic N-terminal domain which contains trafficking motifs that regulate the subcellular distribution of IRAP-containing vesicles (Fig. 1)^1^.

A diverse series of names have been used for IRAP including oxytocinase^2^, placental leucine aminopeptidase^3^ and the angiotensin type IV receptor^4^, reflecting the multifunctional nature of the protein and its broad tissue distribution throughout the body. For example, it has been implicated in a range of physiological processes including regulating the pregnancy hormone, oxytocin, to prevent the onset of premature labour^2^ and trimming peptides for antigen cross presentation in dendritic cells as part of the adaptive immune response^5^. Growing evidence suggests IRAP may also have a prominent role in disease, with global gene deletion of the enzyme in mice providing protection against ischemic stroke damage^6^, seizures^7^ and diet-induced obesity^8^. Similar protection is observed following inhibition of the catalytic activity of IRAP with specific inhibitors^9^, which show promise as potential therapeutic agents for multiple disease and injured states. Interestingly, the tissue expression of IRAP is upregulated in various pathological states including atherosclerosis^10^ and following balloon injury of carotid arteries^11^, suggesting it may play a role in the pathogenesis or progression of these diseases. This upregulation also extends to different forms of cancer, with high IRAP protein expression seen in cancers of the breast, colon, endometrium, lymph, prostate, skin and thyroid^12^. Together, these findings support the notion that IRAP is a potential therapeutic target in these diseases.

Whilst IRAP is predominantly expressed in its membrane bound form, Rogi, et al.^13^ were the first to report a soluble form of the enzyme that can be detected in the serum of pregnant women. This soluble IRAP (sIRAP) was initially cloned from a human placental library and subsequently identified as a major enzyme present in maternal serum^13^. It is believed to be generated by cleavage of the extracellular domain (Fig. 1) between Phe154 and Ala155 by a secretase in the ‘a disintegrin and metalloprotease domain’ (ADAM) family that has a molecular weight of ~ 150 kDa observed in Western blots^14^. IRAP is thought to be secreted from the apical membrane of placental syncytiotrophoblast cells following the detection of sIRAP in the conditioned media of cultured placental tissue^14^. This secretion of sIRAP from the placenta correlates with increases in circulating oxytocin, which is a substrate of IRAP^2^. Therefore, by regulating oxytocin levels, the soluble form of IRAP in the circulation is thought to play a role in pregnancy.

Based on the findings of increased tissue expression of IRAP in specific disease states and the presence of a soluble, secreted form of the protein in human circulation, we propose that sIRAP may serve as a potential biomarker. However, there are currently no reliable methods to measure and quantify secreted IRAP and validate its potential as a disease biomarker. Commercially available anti-IRAP antibodies are predominantly directed to target the cytoplasmic N-terminal domain whilst the specificity of the available ELISA kits has not been validated with no information provided on the binding region of the antibodies or the source of IRAP used to develop the assay. Therefore, the aim of this study was to develop a series of specific monoclonal antibodies targeting the C-terminal domain of IRAP for use in a sandwich ELISA to measure and quantify sIRAP levels in human plasma.

## Results

### Generation and characterisation of novel anti-IRAP antibodies

Antibodies that recognise the soluble C-terminal domain of human IRAP were successfully generated by Monash Antibody Technologies Facility (MATF) using hybridoma technology. Four distinct clones (RF7, RB9, RH3, RG4) were selected for expansion and purification based on their binding profiles with high specificity for sIRAP over other related aminopeptidases.

**Figure 2:**
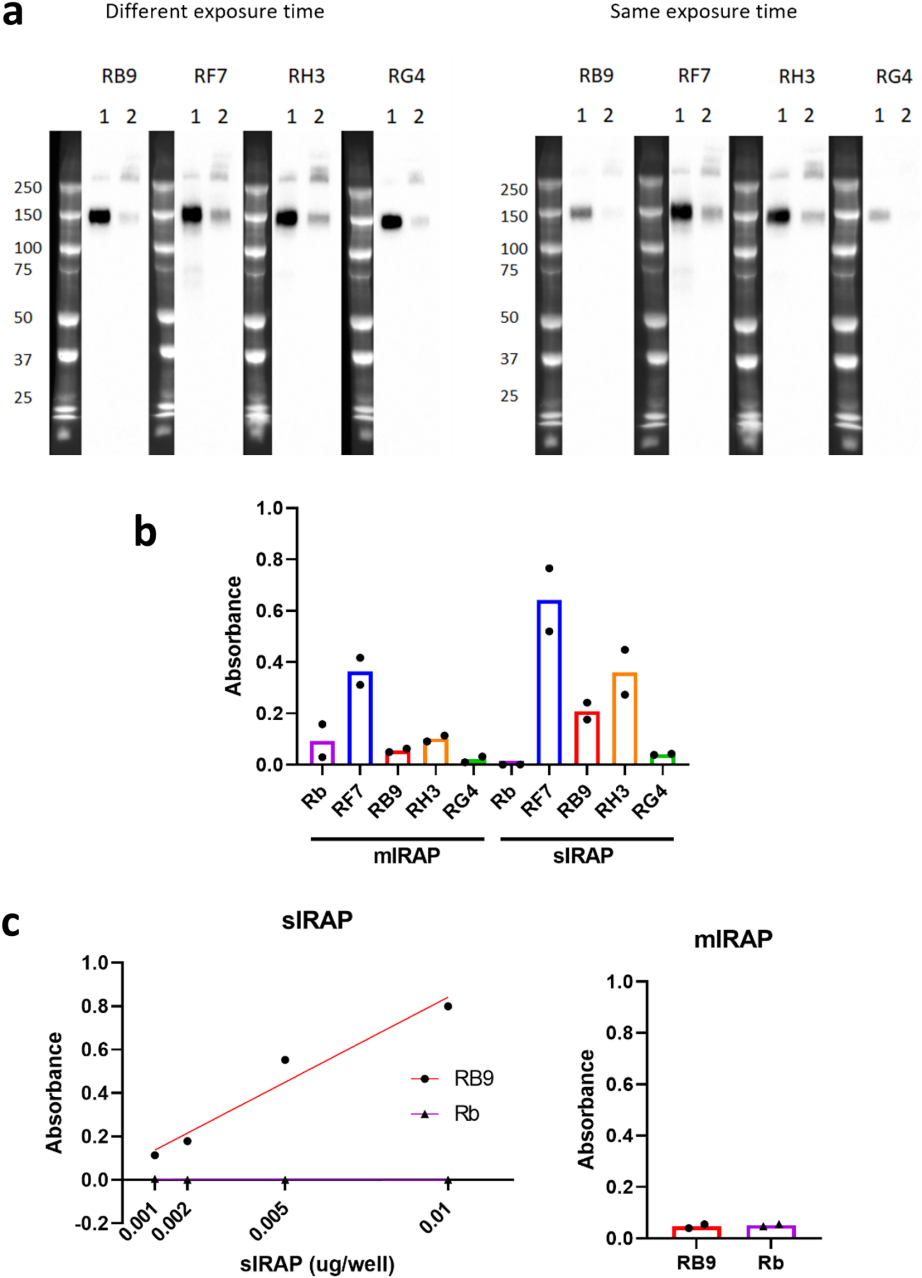
The novel anti-IRAP antibodies can specifically detect human IRAP. (**a**) Representative Western blots (uncropped 12 lanes in same gel) showing binding of the novel mouse anti-IRAP antibodies (RB9, RF7, RH3, RG4; 0.5 µg/µl) to (1) full length IRAP derived from membrane preparations of HEK293T cells transiently overexpressing human IRAP (0.01 µg protein/µl) or (2) purified soluble human IRAP (0.001 µg/µl). The lanes for each antibody were imaged at either different exposure times or at the same exposure time across all antibodies (n = 3). Original, uncropped blots are provided in Supplementary Fig. 1. (**b**) The mouse anti-IRAP antibodies (1 µg/well) and the commercially available rabbit anti-IRAP antibody (Rb; 1:500 concentration) were tested for binding to mIRAP (10 µg protein/well) or purified sIRAP (0.001 µg/well) using an indirect ELISA. Data presented is the mean from two separate experiments performed in duplicate (n = 2). (**c**) The mouse RB9 clone (1 µg/well; red) and the rabbit antibody (1:500; purple) were tested against decreasing concentrations of purified sIRAP (0.001–0.01 µg/well) and mIRAP (10 µg protein/well). Simple linear regression was conducted (R^2^ = 0.94). Data presented is the mean of absorbance readings from one experiment performed in duplicate (n = 1).

To further validate the specificity of the antibodies to the different forms of IRAP, Western blotting was conducted. Recombinant soluble human IRAP, prepared by CSIRO and referred to as purified sIRAP, was used as the source of sIRAP in all experiments and membrane preparations of HEK293T cells transiently transfected to overexpress IRAP were used as the source of full length IRAP (mIRAP). All four mouse antibodies successfully bound to both mIRAP and sIRAP with a specific band at ~ 150–160 kDa (Fig. 2a). Dimerised forms of both mIRAP and sIRAP were also observed with sIRAP displaying a greater tendency to form dimers compared with monomers (molecular weight > 250 kDa; Fig. 2a). Notably, differences in the binding of an antibody to mIRAP and sIRAP may be due to differences in the amount of IRAP loaded given the exact concentration of IRAP in the membrane preparation is unknown. When images were captured at the same exposure time, a difference in the intensity of the bands of each antibody can also be seen. This suggests they have different binding properties and likely bind to different epitopes of IRAP.

The antibodies were then tested in an indirect ELISA to confirm whether there were any differences in potential binding to IRAP. RF7 binds to mIRAP and sIRAP more effectively than the other antibodies, followed by RH3 and RB9 (Fig. 2b). Whilst the antibodies appear to be less effective at binding to mIRAP, this is likely due to the fact that only 5–10% of the total protein in the membrane preparation used as the source of full-length IRAP is actually IRAP.

To determine the lowest detection range in an indirect ELISA of one of the antibodies, RB9 was tested against decreasing purified sIRAP concentrations. Binding of RB9 to sIRAP was detectable in the nanogram range (0.001 µg/well; Fig. 2c). Notably, RB9 produced equivalent readings between purified sIRAP at 0.001 µg/well and mIRAP at 10 µg/well (Fig. 2c). Based on these findings, RB9 and likely the other highly specific antibodies, can detect low levels of sIRAP in the nanogram detection range.

**Figure 3:**
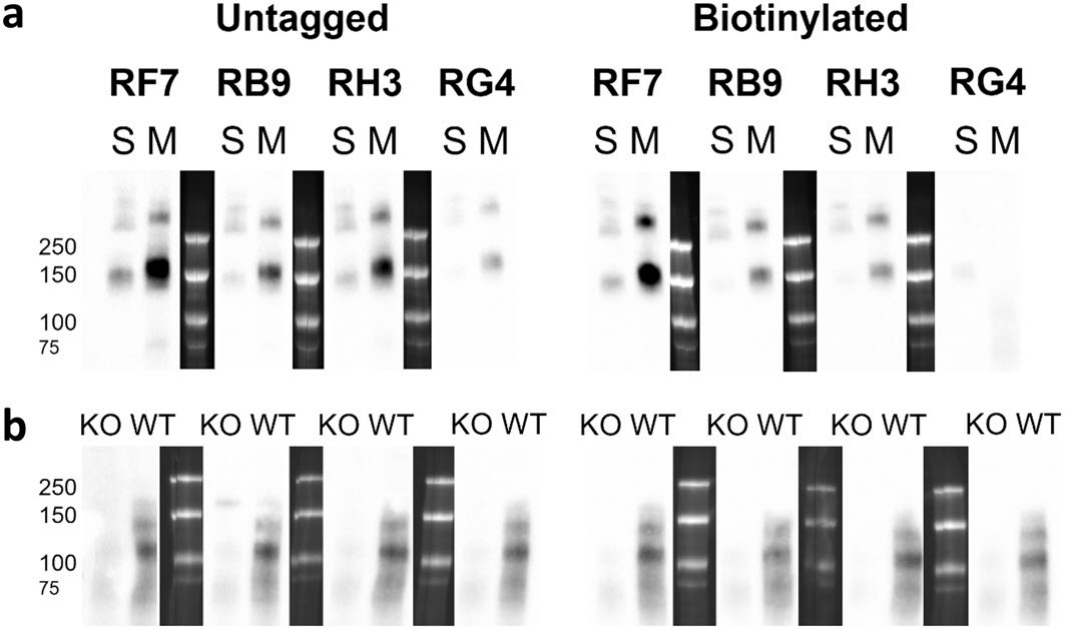
Specificity of the subcloned antibodies for IRAP. Western blots of the untagged and biotinylated newly subcloned monoclonal anti-IRAP antibodies RF7, RB9, RH3 and RG4 (0.0005 µg/µl) against either (**a**) purified soluble human IRAP (S; 0.001 µg/well) and full length human IRAP enriched membrane preparations of HEK293T cells transiently transfected with human IRAP cDNA (M; 0.1 µg proteins/well) or (**b**) protein lysate from cardiac tissue of 10-week old male IRAP KO or WT mice (20 µg protein/well), n = 1. All blots are imaged for the same exposure time. Original, uncropped blots are provided in Supplementary Fig. 2.

Due to these promising results, the hybridoma cell lines were subcloned and the antibodies purified and biotinylated by MATF. The specificity of the untagged and biotinylated antibodies was then tested using Western blots. All eight antibodies produced a band at ~ 150 kDa for purified sIRAP and at ~ 165 kDa for mIRAP, except biotinylated RG4 which did not bind well to either form of the protein (Fig. 3a; Supplementary Fig. 1). Notably, IRAP dimers are also evident in both purified sIRAP and mIRAP preparations. IRAP isoforms ranging from ~ 120–165 kDa were also detected in protein lysates of cardiac tissue from male wildtype (WT), but not IRAP knockout (KO) mice with all eight antibodies (Fig. 3b). Overall, the detection of a band of the correct size as purified sIRAP and the absence of specific IRAP bands in tissue from IRAP KO mice, demonstrate that all the subcloned antibodies are highly specific for IRAP.

**Figure 4:**
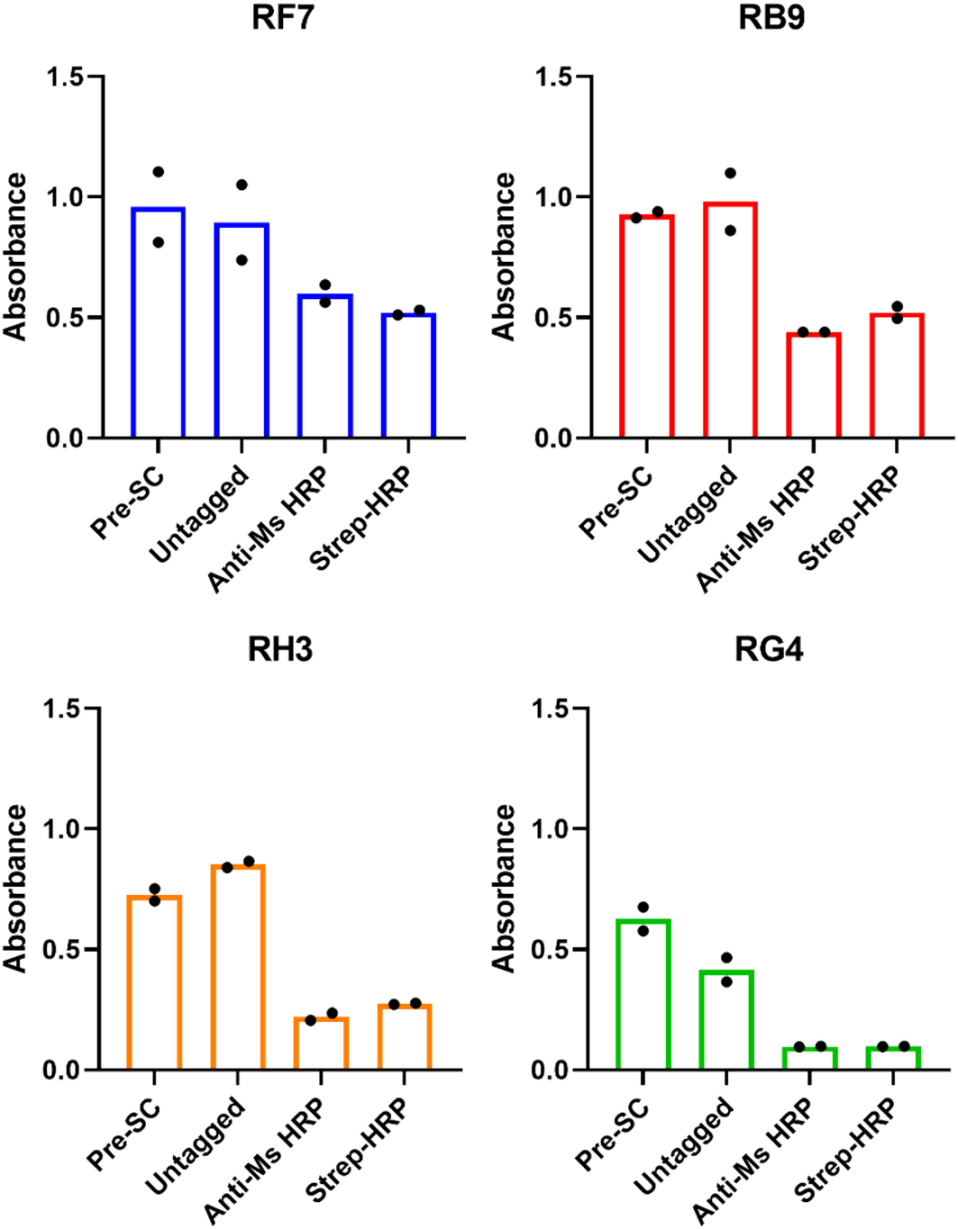
Initial screen of newly subcloned antibodies. An indirect ELISA using purified sIRAP (4 µg/ml) was conducted to compare the pre-subcloned (pre-SC) antibodies with the newly subcloned antibodies which were either untagged or biotinylated (detected using anti-Ms-HRP secondary antibody or streptavidin-HRP). All antibodies were used at 1 µg/well. The experiment was performed in duplicate (each dot is raw duplicate value, bar represents the mean, n = 1).

Lastly, the ability of the highly pure and tagged antibodies to maintain their binding to purified sIRAP was tested in an indirect ELISA and the outcome was compared to the pre-subcloned antibodies. All four untagged, subcloned antibodies maintained their ability to bind to sIRAP (4 µg/ml; Fig. 4). However, the biotinylated antibodies which were detected using either an anti-mouse HRP secondary antibody or streptavidin-HRP, had varying decreases in their binding ability compared to their untagged counterparts (Fig. 4). In particular, biotinylated RH3 displayed consistently decreased sIRAP binding compared to the untagged RH3 (Fig. 4). Overall, the subcloned antibodies successfully bound to IRAP and display potential for use in an IRAP sandwich ELISA which, unlike the indirect ELISA, should have improved sensitivity and allows for quantification of the amount of IRAP in an unknown sample using an sIRAP standard curve.

### Development of sandwich ELISA

**Figure 5:**
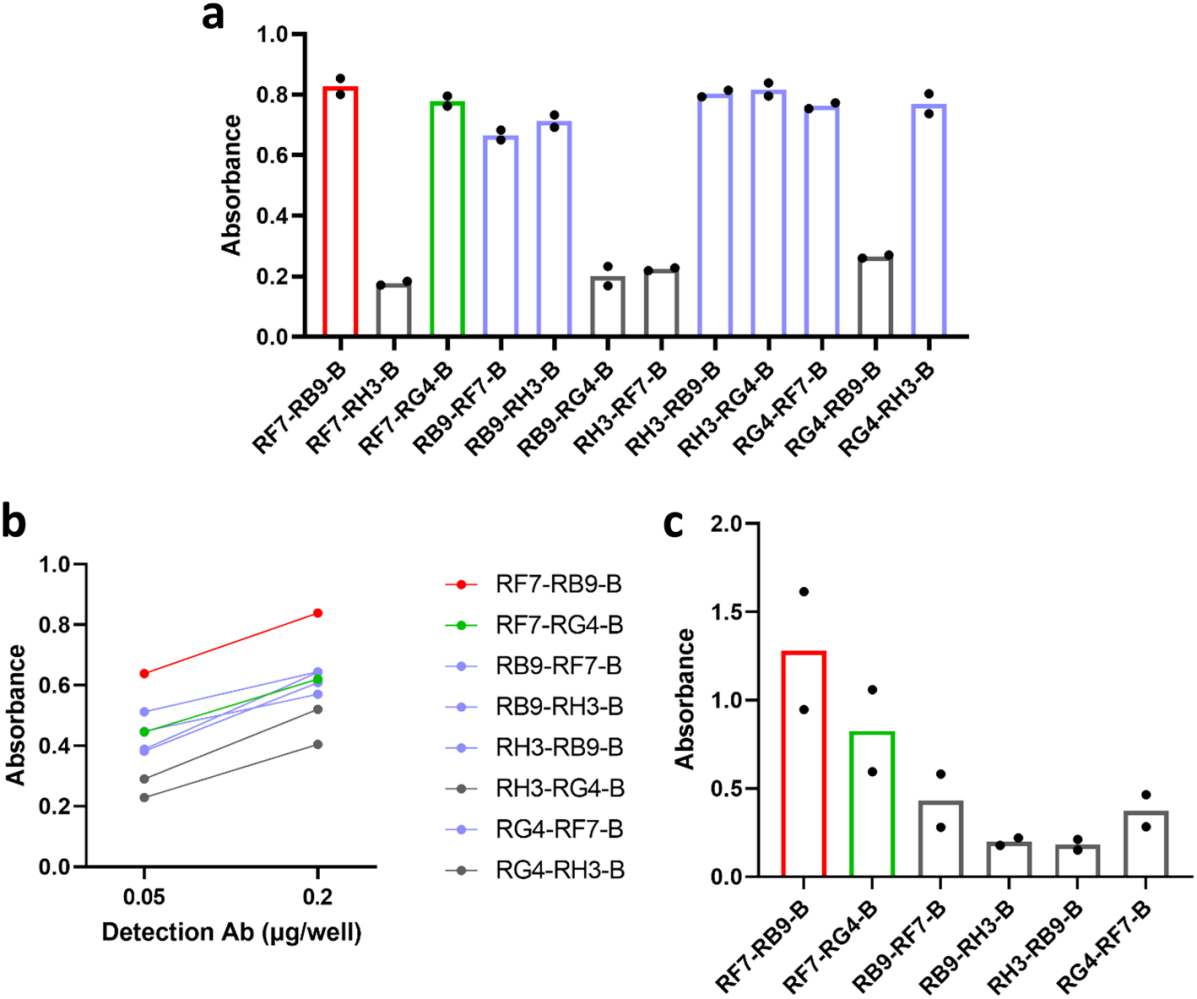
RF7-RB9-B and RF7-RG4-B are the top performing antibody combinations. (**a**) All 12 potential combinations of capture (2 µg/well) and detection (1 µg/well) antibodies were tested in a sandwich ELISA using purified sIRAP (4 µg/ml). (**b**) For the top eight combinations, lower concentrations of detection antibody (0.05 & 0.2 µg/well) were used. (**c**) For the top 6 combinations, an even lower capture (0.2 µg/well) and detection (0.01 µg/well) antibody concentration was used. The top performing combinations, RF7-RB9-B and RF7-RG4-B are displayed in red and green, respectively, whilst the top combinations that progressed to the subsequent assay are displayed in blue. Combinations excluded from subsequent testing are displayed in grey. Each experiment was performed in duplicate (each dot in bar graphs show raw duplicate values, bar represents the mean, n = 1).

The first step in the development of a sensitive IRAP sandwich ELISA was to determine the best capture and detection antibody combination to use. All 12 possible capture (2 µg/well) and detection (1 µg/well) antibody combinations were initially screened against purified sIRAP (4 µg/ml). Eight combinations successfully detected sIRAP while the remaining four were excluded from further experimentation (Fig. 5a). Interestingly, the combination of RF7 and RH3 and the combination of RB9 and RG4 consistently failed to detect sIRAP (Fig. 5a). These observations suggest that these antibody pairs likely bind to epitopes in a similar region and their combined use in a sandwich ELISA is therefore not viable. The top eight antibody combinations from this initial screen were subsequently tested at lower capture (0.1 & 0.5 µg/well) and detection (0.05 & 0.2 µg/well) antibody concentrations. Decreasing the capture antibody to 0.1 µg/well resulted in a loss of sIRAP detection, highlighting the importance of maintaining high capture antibody concentrations. Therefore, only data at the capture antibody concentration of 0.5 µg/well is presented. RF7-RB9-B was the top performing antibody combination (Fig. 5b). The top six combinations from this experiment progressed to the next stage of testing where capture (0.2 µg/well) and detection (0.01 µg/well) antibody concentrations were further decreased. Again, RF7-RB9-B outperformed all other antibody combinations (Fig. 5c). The top two combinations, RF7-RB9-B and RF7-RG4-B were both selected to progress in the development of the IRAP sandwich ELISA.

**Figure 6:**
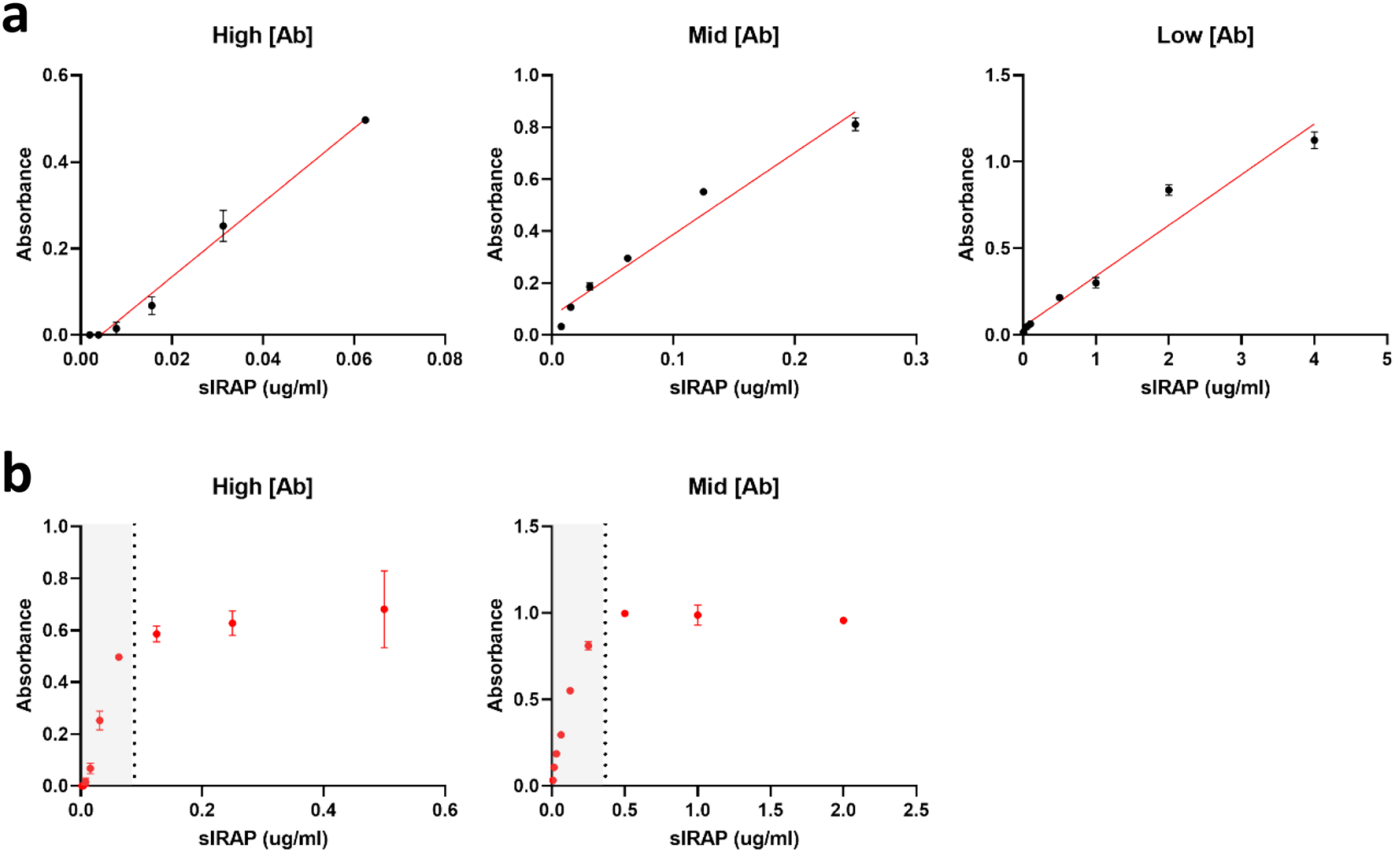
Optimising antibody concentrations. (**a**) Standard curves at varying concentrations of purified sIRAP (µg/ml) were determined at high (RF7 = 2 µg/well, RB9-B = 1 µg/well), mid (RF7 = 0.5 µg/well, RB9 = B = 0.25 µg/well) and low (RF7 = 0.2 µg/well, RB9-B = 0.1 µg/well) antibody concentrations. (**b**) The absorbance readings in sandwich ELISAs plateaued over time at high and mid antibody concentrations. Simple linear regression was conducted to generate the standard curves. Data presented is the mean ± SEM of absorbance readings from one experiment performed in duplicate (n = 1).

The next step in the development of the sandwich ELISA was to determine the optimal antibody concentrations that could detect the low levels of sIRAP (nanogram range) expected to be present in plasma. The RF7-RB9-B combination was used for this optimisation. As expected, the purified sIRAP detection range was highly dependent on the concentration of antibodies used. At low capture (0.2 µg/well) and detection (0.1 µg/well) antibody concentrations, purified sIRAP could only be accurately detected down to a concentration of 0.5 µg/ml (Fig. 6a). Given the levels of sIRAP in plasma are expected to be in the lower nanogram range, antibody concentrations were increased to improve sensitivity of the detection. At both the mid and high capture (0.5 & 2 µg/well) and detection (0.25 & 1 µg/well) antibody concentrations tested, low nanogram levels (~ 10 ng/ml) of purified sIRAP could be accurately and reliably detected (Fig. 6a). However, at the highest tested antibody concentrations, the standard curve quickly saturated at the relatively low antigen concentration of 125 ng/ml (Fig. 6b). Therefore, the mid antibody concentrations were selected to measure sIRAP in plasma given the detection of the protein fell within a broader linear range of the standard curve.

### Validation of optimised sandwich ELISA

**Figure 7:**
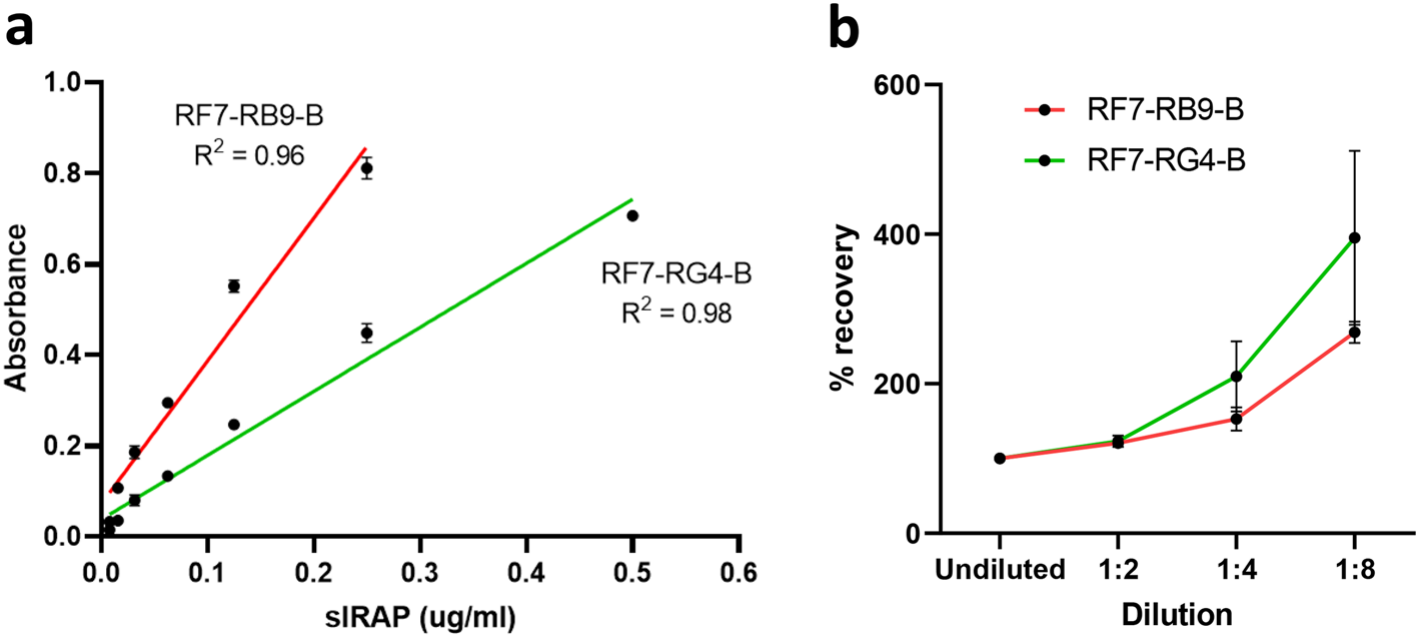
Determining the detection range and linearity of the sandwich ELISAs. (**a**) A sandwich ELISA at serially diluted concentrations of purified sIRAP (0.0078—0.5 µg/ml) was conducted using both RF7-RB9-B (red) and RF7-RG4-B (green) antibody combinations at the optimised capture (0.5 µg/well) and detection (0.25 µg/well) antibody concentrations. Simple linear regression was conducted to generate the standard curve. Data presented is the mean ± SEM of absorbance readings from one representative experiment performed in duplicate (n = 3). (**b**) The linearity of the RF7-RB9-B (red) and RF7-RG4-B (green) ELISAs at the optimised concentrations was determined by calculating the % recovery of pooled control human plasma spiked with 0.25 µg/ml purified sIRAP at serial dilutions. Data is presented as the mean ± SEM from 3 experiments performed in triplicate (n = 3).

To determine the specific detection range for both selected antibody combinations at the optimised concentrations, sandwich ELISAs were conducted at progressively decreasing concentrations of purified sIRAP. The concentrations of sIRAP in the nanogram range could be detected with both RF7-RB9-B (16–250 ng/ml; n = 3) and RF7-RG4-B (31–500 ng/ml; n = 3; Fig. 7a).

The sensitivity, or lower limit of detection, of the sandwich ELISAs was also calculated by determining the lowest measurable concentration that is statistically different from the zero standard. Twelve replicates of zero standards were used in each experiment. The sensitivity of the ELISAs was calculated to be 9.1 ± 3.6 ng/ml for RF7-RB9-B (n = 3) and 9.6 ± 4.4 ng/ml for RF7-RG4-B (n = 3).

The precision of the ELISAs was determined by measuring the intra- and inter-assay variability. The coefficient of variance (%CV) within an experiment (12 replicates of purified sIRAP at 0.25 µg/ml) was calculated to be 8.32 ± 2.16% for RF7-RB9-B (n = 3) and 8.10 ± 0.98% for RF7-RG4-B (n = 4). The %CV between experiments conducted on separate days with 12 replicates each of 0.25 µg/ml purified sIRAP was 14.72% for RF7-RB9-B (n = 3) and 26.55% for RF7-RG4-B (n = 4). The inter-assay variability is slightly higher than expected, possibly due to variability in the reaction time. However, the inclusion of a standard curve on every plate will minimise the effects of variability in assay conditions when testing human samples.

The spike recovery of each ELISA was assessed by spiking a known concentration of purified sIRAP into pooled control plasma and calculating if the recovered value significantly differs from the expected concentration. This was determined to be 70.68 ± 3.04% for RF7-RB9-B (n = 3) and 50.27 ± 0.96% for RF7-RG4-B (n = 3). These values indicate that there is possible interference by binding proteins in the plasma that mask the detection of purified sIRAP. To ensure consistency in the interference between different samples, the purified sIRAP in the standard curve and human plasma samples will be prepared under identical conditions (i.e. same concentrations of buffer:plasma).

Finally, the linearity was assessed by comparing the corrected values of serially diluted pooled control plasma spiked with a known concentration of purified sIRAP. Values between 80–120% are generally accepted as linear. The % recovery of dilutions greater than 1:2 were found to be above the accepted maximum recovery of 120% for both antibody combinations (Fig. 7b). Therefore, in future assays, samples will not be diluted with factors greater than 1:2.

Given the concentration of sIRAP in diluted plasma is expected to fall within the detection range and parameters such as the intra-assay variability have been validated, the two optimised sandwich ELISAs present as a highly specific and sensitive tools to measure circulating sIRAP.

### Further validation using human plasma

To validate the optimised sandwich ELISA can detect endogenously expressed sIRAP in human plasma, samples from women at various stages of pregnancy were tested given that IRAP expression is known to increase in the circulation throughout gestation and is thought to peak at term^2^. Plasma samples were collected from non-pregnant, young female volunteers (n = 6). Plasma samples were also collected from pregnant women at 28-, 36-weeks gestation and at term from the Mercy Hospital for Women and these samples were pooled in separate batches (n = 5–6). It is important to note that there is a possibility that the term plasma samples were from pregnancies where borderline growth restriction of the foetus had been documented.

**Figure 8:**
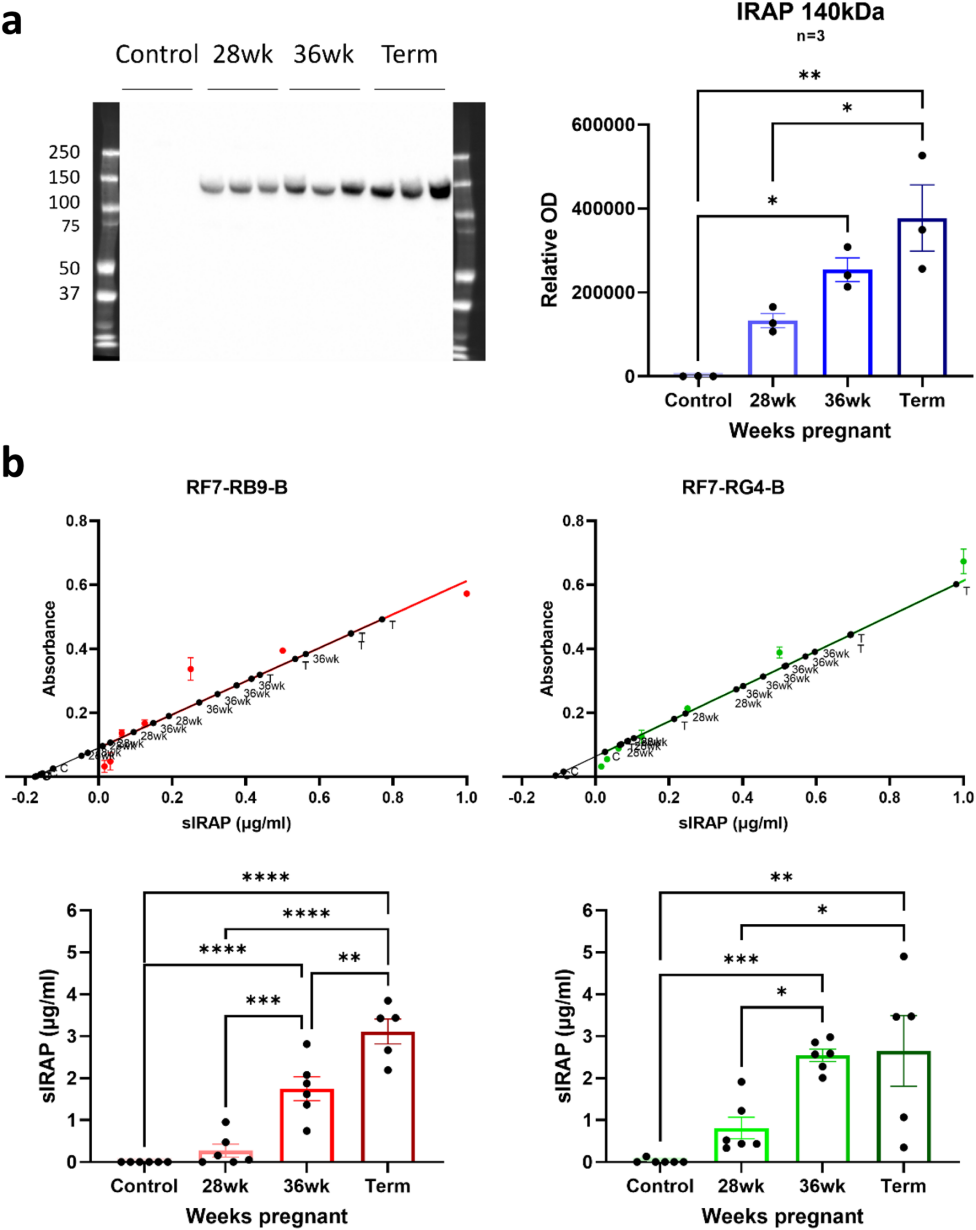
Detection of increases in sIRAP expression in human plasma throughout the later stages of pregnancy. (**a**) Representative Western blot and quantification of the optical density (OD) of sIRAP (140–150 kDa) in plasma from healthy controls and women at later stages of pregnancy (28- & 36-weeks and full term; diluted 1:10), detected using the subcloned RF7 anti-IRAP antibody (0.0005 µg/µl). Control plasma is from three different participants and pregnant plasma is three different batches of pooled samples (n = 3). Data is expressed as mean ± SEM, was corrected for total protein concentration and analysed using a one-way ANOVA with Tukey’s post-hoc test, *p < 0.05, **p < 0.01 compared to control. (**b**) Sandwich ELISAs using RF7-RB9-B (red) and RF7-RG4-B (green) antibody combinations was conducted to measure concentrations of IRAP in plasma from control and pregnant women (28- & 36-weeks & term) using a purified sIRAP standard curve (µg/ml). All samples were diluted 1:5. Data was analysed using simple linear regression analysis to interpolate the concentrations of each sample and a one-way ANOVA with Tukeys’s post-hoc test to compare groups to the control, *p < 0.05, **p < 0.01, ***p < 0.001, ****p < 0.0001 vs control, n = 5–6. All data was corrected for the negative control and is presented as mean ± SEM.

Firstly, the ability of the RF7 monoclonal anti-IRAP antibody (ELISA capture antibody) to detect IRAP in select pregnant plasma samples was investigated by Western blot analysis. There was a significant increase (p = 0.0011, one-way ANOVA) in IRAP expression in the third trimester of pregnancy with a specific band at the expected molecular weight for sIRAP (140–150 kDa; Fig. 8a). Notably, no bands were detected using a commercially available, rabbit anti-IRAP antibody which binds to the N-terminal domain, confirming that the RF7 antibody is indeed detecting sIRAP. Overall, this preliminary finding supports previous literature^2^ and confirms the plasma samples are suitable for use to validate the utility of the sandwich ELISAs in measuring sIRAP in human samples.

The plasma samples from pregnant women were then tested in the IRAP sandwich ELISAs. Surprisingly, all plasma samples, including that from the later stages of pregnancy, produced similar absorbance values which were indistinguishable from the negative control in the ELISA using the RF7-RB9-B antibody combination. Given that the Western blots using the RF7 capture antibody detected increases in sIRAP in samples from the later stages of pregnancy, the lack of positive signal in the ELISA suggests sample matrix interference with the binding of the biotinylated detection antibody (RB9-B). This may be due to the propensity of this isoform of IRAP to adopt different conformations, form homo- or hetero-dimers, or interact with binding proteins in plasma. Therefore, to disrupt the conformation of IRAP and expose the antibody binding sites, samples were tested under different denaturing & reducing conditions (see methods). For the RF7-RB9-B antibody combination, samples prepared in 2.5% SDS/3% BSA/PBS and heated at 70°C for 5 min was optimal for the detection and quantification of sIRAP in pregnant plasma samples as well as the recombinant sIRAP in the standard curve. However, for the RF7-RG4-B antibody combination, heating at 70°C for 5 min alone was sufficient for the detection of sIRAP. Using these optimised conditions, significant increases in the levels of sIRAP were detected in plasma from women at the later stages of pregnancy with both antibody combinations (RF7-RB9-B *p* < 0.0001, RF7-RG4-B p = 0.001; one-way ANOVA). A consistent level of expression around 3 µg/ml was detected in the term samples (Fig. 8b). Importantly, the detection of sIRAP in pregnant plasma using the novel sandwich ELISAs mirrors the results obtained from Western blot experiments, confirming the analytical validity of the IRAP ELISAs.

## Discussion

Biological markers or biomarkers, are defined biological characteristics that are used to indicate normal physiological processes, pathogenic processes or responses to an intervention^15^. There is increasing awareness that the discovery of new biomarkers goes hand in hand with the identification of new therapeutic targets as they have the potential to markedly accelerate all phases of drug development and improve clinical outcomes.

IRAP is a multifaceted enzyme and is currently being pursued as a therapeutic target for conditions such as ischemic stroke^6,9^ and cardiovascular disease^16^. Excitingly, it possesses a number of unique properties which also position it as a potential biomarker for specific conditions. For example, IRAP is a type II transmembrane protein and the extracellular domain of the enzyme can be cleaved and secreted into the blood stream or interstitial space in humans. Changes in plasma IRAP levels may also correlate with specific physiological or pathological conditions. However, the commercially available ELISA kits for the detection of IRAP have not been validated nor have the antibodies been shown to bind specifically to the soluble portion of the enzyme. Therefore, a detection method with high analytical validity was developed in this study which could be used to explore the potential for IRAP to serve as a disease biomarker.

Firstly, novel anti-IRAP antibodies which are directed towards the C-terminal domain of the enzyme were generated and characterised. The specificity of the antibodies for IRAP was confirmed by the detection on a Western blot of a 165 kDa band corresponding to full length IRAP and a 150 kDa band corresponding to sIRAP. Importantly, there was also a clear absence of a band in protein lysates of cardiac tissue from IRAP KO mice. IRAP has the propensity to form homodimers, which were also detected by the antibodies.

A sandwich ELISA was then developed which involved optimisation of a range of factors including the antibody combination, antibody concentration, denaturing & reducing conditions. Parameters such as the detection range, sensitivity, intra- and inter-assay variability, spike recovery and linearity were also assessed. Collectively, these optimised conditions enabled the detection of nanogram levels of purified sIRAP using two different antibody combinations which is a great strength of this study. To further validate that the optimised sandwich ELISAs can detect sIRAP, human plasma samples from women at various stages of pregnancy were tested for sIRAP expression which is known to increase in the circulation throughout gestation^2^. Excitingly, the ELISAs with different anti-IRAP antibody combinations can detect increasing levels of sIRAP in plasma from pregnant women, which correlated with gestational period, validating its potential clinical utility in the detection and quantification of sIRAP in human plasma.

Recently, the expression of sIRAP in the serum has been proposed as a potential biomarker of pre-diabetes^17,18^. Patients with insulin resistance displayed slightly lower levels of sIRAP in the serum compared to euglycemic controls^17^. Unlike healthy individuals where IRAP levels increase following a glucose challenge, IRAP in the serum of insulin-resistant diabetic patients remained unaltered^17^. A major limitation of this study is the sample size of two. Another study by Mostafa, et al.^18^ also reported a decrease in sIRAP levels in type II diabetic patients compared to euglycemic controls leading the authors to propose the utility of sIRAP as a potential biomarker of pre-diabetes. However, the reliability of the ELISAs used and their ability to detect the soluble from of the enzyme has not been demonstrated. In the study by Trocme, et al.^17^, the reported size of the recombinant sIRAP bands in their Western blots does not correspond to the known size of sIRAP (~ 150 kDa), appearing instead at as a 180 kDa band which does not represent full-length (165 kDa) or dimerised (> 250 kDa) IRAP. In the few other reported studies measuring circulating IRAP as a biomarker for pre-diabetes^18^, gestational diabetes^19^, preeclampsia^20^ and prostate cancer^21^, commercially available ELISA kits were used, but no information was provided for the ELISAs on the region of IRAP the antibodies are directed against or validation of their specificity.

An interesting point of difference between the two reports on the use of IRAP as a biomarker of insulin resistance is the level of sIRAP detected in the serum of healthy control participants. Whilst Trocme, et al.^17^ reported relatively high levels of sIRAP in control human serum of 101 ± 16 μg/ml (from 2 individuals), the study by Mostafa, et al.^18^ only detected sIRAP levels in the 96 ± 14 ng/ml range (from 60 individuals), almost 1000-fold difference. In the current study, levels of sIRAP in control human plasma were below the detection limit in Western blots and the sandwich ELISAs which can accurately detect down to 16 ng/ml of purified sIRAP. This finding of undetectable levels of sIRAP expression in healthy control plasma is strengthened by the use of two different antibody combinations which are predicted to bind to distinct IRAP epitopes. This stark difference in the reported levels of sIRAP in healthy controls in all three studies questions the analytical validity of some of the assays. Notably, the age and sex of the control participants was not stated in the study by Trocme, et al.^17^ which may explain some of the differences in sIRAP levels. Low levels of sIRAP expression at ~ 0.06 ng/ml and ~ 2 ng/ml were also reported in other studies in pregnant women between 20–22 and 25–35 weeks gestation, respectively^19,20^. However, since measurements in non-pregnant, healthy controls were missing in these studies and in all other studies examining circulating IRAP^21–24^, it is difficult to draw any conclusions on IRAP levels in healthy humans.

In summary, the RF7 antibody that was used as the capture antibody in the ELISAs in the current study, produces a single specific band representing sIRAP in human plasma (140–150 kDa) on a Western blot with the specificity of the antibody validated by the absence of specific bands in tissue from IRAP KO mice. Additionally, the RF7 antibody can detect increasing sIRAP expression in the plasma of pregnant women which correlated with advancing stages of gestation, demonstrated by both Western blot analysis as well as the sandwich ELISAs. Therefore, the ELISAs developed in the current study have the potential to accurately measure IRAP levels in plasma given (1) the high specificity of our monoclonal antibodies for sIRAP, (2) the inclusion of appropriate positive and negative controls and (3) the confirmation of the analytical validity of the ELISA by measuring human samples where the sIRAP levels were known to be regulated.

The next phase in the development of the sandwich ELISA is the validation of IRAP as a biomarker for certain disease states. Experiments examining the mechanisms underlying IRAP secretion and the specific cell types involved are also crucial to strengthen the concept of IRAP as a biomarker for certain diseases. Together, the use of the sandwich ELISA to quantify IRAP and its association with diseased conditions could significantly accelerate clinical trials testing IRAP inhibitors by identifying suitable trial participants, assisting in the diagnosis of disease and monitoring the efficacy and safety of therapeutic intervention. Overall, this could markedly improve patient outcomes with the early detection of disease preventing subsequent organ damage and dysfunction.

## Methods

### Generation of monoclonal IRAP antibodies

Antibodies directed towards the soluble C-terminal domain of human IRAP were generated using hybridoma technology by the Monash Antibody Technologies Facility (MATF, Monash University, Australia). Briefly, six mice with global gene deletion of IRAP were immunised 3 times at two-week intervals (i.p.) with a combination of 16 µg of recombinant soluble human IRAP protein and immune adjuvant (Sigma #S6322) with methylated CpG until a strong serum antibody titre was reached. The spleens of the mice were fused with SP2/0-Ag14 myeloma cells using polyethylene glycol to generate antibody-producing hybridomas. Hybridoma colonies were grown for 10 days at which point the number of hybridoma colonies was determined and after a further 3 days incubation, an aliquot of antibody supernatant was taken for screening. The cell culture supernatants were screened for antibodies that bound to soluble human IRAP by antigen microarray and these results were confirmed by antigen ELISA. The antibody containing supernatants were also assessed by Western blot to determine the specificity for IRAP over other related aminopeptidases including aminopeptidase N, endoplasmic reticulum aminopeptidase I and II. Based on the binding profiles, four hybridoma clones were chosen for further analysis (clones RF7, RG4, RB9 and RH3). The cells were expanded and antibody was purified from the supernatant using Protein A/G affinity chromatography by MATF. Later, the antibodies were subcloned and biotinylated by MATF.

### Sources of IRAP used in experiments

Recombinant soluble human IRAP (referred to as purified sIRAP) was generated by CSIRO (Melbourne, Australia). The IRAP coding-region templates were synthesised by Genscript, codon-optimised for transcription/translation in human cells and subcloned into the mammalian expression vector, pCAGGS, for preparation of the plasmid. For the production of sIRAP, the N-terminal 131 amino acids encoding the cytoplasmic and transmembrane-spanning sequences were removed and replaced by the mouse interleukin-3 (IL-3) SP, followed by four amino acids (ASIS) corresponding to the mature IL-3 N-terminus, the IRAP extracellular domain (894 amino acids) and a C-terminal with a mFlag epitope tag (DYKDDDDK). One microgram of plasmid was transfected into freestyle HEK 293-F cells at a ratio of 1:3 [DNA:PEI]. The cells were maintained for 7–9 days after which they were harvested and recombinant IRAP was purified using anion-exchange chromatography followed by gel filtration on a Superdex S200 26/60 column.

Protein lysates prepared from cardiac tissue of 10-week old male WT and IRAP KO mice were used to test the specificity of the novel antibodies. Briefly, the tissue was homogenised in 300–500 µl of 1.5 × Laemmli buffer (diluted from 5 × Laemmli buffer (25% glycerol, 7.5% SDS, 250 mM Tris–HCl pH 8.0, 0.25 mg/ml bromophenol blue)) containing 2-mercaptoethanol and then sonicated for 6 s, 3 times. The protein was heated in a water bath at 37 °C for 10 min before being centrifuged at 13,000 rpm for 10 min at 4 °C. The protein-containing supernatant was transferred to new tubes.

All experiments involving animals were approved by the Monash University Animal Ethics Committee and conducted in accordance with the National Health and Medical Research Council’s, *Australian code for the care and use of animals for scientific purposes* and the ARRIVE guidelines.

Membrane preparations of HEK293T cells (ATCC #CRL-3216) transiently transfected with the expression vector, pCI containing full length cDNA for human IRAP were used as an enriched source of full length IRAP (mIRAP), following the methodology previously described by Lew et al.^25^.

The collection of plasma from healthy adults was approved by the Monash University Human Research Ethics Committee (#37,732) and conducted in accordance with the NHMRC guidelines, National Statement on Ethical Conduct in Human Research 2007. Informed consent was obtained from all participants. Separate batches of pooled plasma from women at 28- and 36-weeks of gestation and full-term were kindly provided by Professor Natalie Hannan, Mercy Hospital for Women. It is worth noting that the term samples were from pregnancies where there was borderline growth restriction of the foetus.

### Indirect ELISAs

All antibody and antigen concentrations used are specified in the results for each individual experiment. Nunc MaxiSorp 96-well ELISA plates were coated with the appropriate antigen diluted in coating buffer (0.1 M Na_2_CO_3_/NaHCO_3_, pH 9.6) overnight (50 μl/well). Following overnight antigen coating, the plates were washed 3 times quickly with 1 × PBS. The antigen was then blocked with 5% skim milk in 1 × PBS for at least 30 min (100 μl/well). After washing with 1 × PBS (3 quick washes), plates were incubated with the appropriate anti-IRAP antibody diluted in 5% skim milk for 1 h (50 μl/well). Wash steps were repeated and the appropriate HRP-conjugated secondary antibody (Agilent-Dako) diluted in 5% skim milk (1:2000) was added for 1 h (50 μl/well). Following washing, TMB substrate (ThermoFisher) was added to each well until the desired colour was achieved (10–15 min; 50 μl/well), at which point the reaction was stopped with 0.2 M H_2_SO_4_ (50 μl/well). The absorbance of each well was measured at 405 nm using a CLARIOstar plate reader (BMG Labtech).

### Sandwich ELISAs

All antibody and antigen concentrations used are specified in the results for each individual experiment. Nunc MaxiSorp 96-well ELISA plates were coated overnight with capture antibody diluted to varying concentrations in coating buffer (0.1 M Na_2_CO_3_/NaHCO_3_, pH 9.6; 100 μl/well). Following the overnight incubation, the plates were washed 3 times quickly with 1 × PBS. The plate coated with the antibody was then blocked with blocking buffer (3% BSA in 1 × PBS) for at least one hour (200 μl/well). After washing with 1 × PBS (three quick washes), plates were incubated for 2 h with the antigen samples diluted to varying concentrations in blocking buffer (100 μl/well). In the experiments using human plasma, the purified sIRAP standard curve was spiked with pooled healthy human plasma to mirror conditions of the diluted test plasma samples. Purified sIRAP standards/plasma samples in the experiments using RF7-RB9-B were denatured in 2.5% SDS in blocking buffer and heated at 70 °C for 5 min. The ELISA conditions were optimised by testing varying concentrations of SDS ± 2-mercaptoethanol or dithiothreitol with heating at 70 or 95°C. After the 2-h incubation and washing with 1 × PBS (3 quick washes), plates were incubated with the biotinylated detection antibody diluted to varying concentrations in blocking buffer for 2 h (100 μl/well). Wash steps were repeated and streptavidin-HRP (R&D Systems) diluted in blocking buffer (1:200) was added for 30 min (100 μl/well). Following washing, TMB substrate (ThermoFisher) was added to each well until the desired colour was achieved (2–15 min; 50 μl/well), at which point the reaction was stopped with 0.2 M H_2_SO_4_ (50 μl/well). The absorbance of each well was measured at 450 nm and corrected for optical imperfections by subtraction of the absorbance at 570 nm. In experiments using human plasma, the absorbance of the negative control containing no antigen was also subtracted from each sample reading.

### Western blotting

To test the specificity of the anti-IRAP antibodies and to validate the protein expression of IRAP in various samples, standard Western blotting was conducted. All samples were diluted in 1.5 × Laemmli buffer (diluted from 5 × Laemmli buffer (25% glycerol, 7.5% SDS, 250 mM Tris–HCl pH 8.0, 0.25 mg/ml bromophenol blue)) and heated at 70 °C for 5 min. Sources of IRAP used include mIRAP (0.01 µg protein/µl or 0.1 μg protein/well), purified sIRAP (0.001 µg/µl or 0.001 μg/well), protein lysates from hearts of male WT or IRAP KO mice (20 μg/well) and human plasma (diluted 1:10). Before loading of samples, 7.5% or 10% gels (15 wells, 1 mm thickness) were prepared using a TGX Stain-free FastCast Acrylamide starter kit (BioRad). Precision Plus Protein Standards Dual Colour (BioRad; 5 μl) and the samples (10 μl) were loaded into the gel and electrophoresed at 150V for ~ 40–50 min in the presence of Tris/glycine/SDS running buffer. Once proteins on the gel were appropriately separated, they were transferred to a Trans-Blot Turbo Mini-size LF PVDF membrane using the Transfer-Blot Turbo transfer system (BioRad). Following transfer, membranes were washed briefly in Tris-buffered saline-tween (TBS-T; 0.1% Tween-20 in 1 × TBS) and then placed on a shaker (70 rpm) in blocking buffer (5% skim milk/TBS-T) for 1 h. The rabbit anti-IRAP antibody (1:2000; Cell Signalling #6918) or the mouse anti-IRAP antibodies (0.0005 µg/ml) were diluted in the same blocking buffer to the appropriate concentration and incubated on membranes overnight on a shaker (70 rpm) at 4 °C. The next day, membranes were washed with TBS-T (3 × 15 min) and then incubated with either goat anti-rabbit HRP or goat anti-mouse HRP (1:2500; Agilent-Dako) diluted in blocking buffer on the shaker for 1 h at room temperature. After washing with TBS-T (3 × 15 min), membranes were developed with Clarity Western ECL substrate (BioRad) for 3 min and imaged using the digital imager ChemiDoc MP imaging system (BioRad). Individual protein bands could be quantified by measuring the optical density (OD) per unit area using ImageLab software (BioRad).

### Statistical analysis

Results are presented as mean ± standard error of the mean (SEM) and statistical analysis was conducted using GraphPad Prism (GraphPad Prism 6 Software Inc.). *p* < 0.05 is considered statistically significant. Simple linear regression was conducted to generate standard curves at increasing sIRAP concentrations. To compare the expression of IRAP throughout gestation in human plasma in sandwich ELISAs and Western blot experiments, one-way ANOVAs with Tukey’s post-hoc test were conducted.

The formulas used to calculate the sensitivity, intra- and inter-assay variability, spike recovery and linearity of the sandwich ELISAs are summarised below.

1 $$ Sensitivity{ }\left( {ng/ml} \right) = mean{ }\,of{ }\,zero{ }\,standard + 2{*}SD{ }\,of{ }\,zero{ }\,standard $$

2 $$ Intra/inter\, assay{ }\,variability{ }\,\left( {\text{\% }} \right) = \left( {\frac{{SD{ }\,of{ }\,replicates}}{{mean{ }\,of{ }\,replicates}}} \right) \times 100 $$

3 $$ Spike{ }\,recovery{ }\,\left( {\text{\% }} \right) = \left( {\frac{{\left[ {plasma{ }\,spiked{ }\,with{ }\,sIRAP} \right]{ } - { }\left[ {plasma} \right]}}{{\left[ {sIRAP} \right]}}} \right) \times 100 $$

4 $$ Linearity \left( \% \right) = \left( {\frac{{\left[ {diluted \,sample} \right] \div dilution\, factor}}{{\left[ {undiluted\, sample} \right]}}} \right) \times 100 $$

### Supplementary Information

Supplementary Information.

## Data Availability

All data generated or analysed are included in this published article and supplementary files. Further data on this study is available from the corresponding author on reasonable request.

## References

[1] Vear A, Gaspari T, Thompson P, Chai SY (2020). Is there an interplay between the functional domains of irap?. Front. Cell. Dev. Biol..

[2] Yamahara N (2000). Placental leucine aminopeptidase/oxytocinase in maternal serum and placenta during normal pregnancy. Life. Sci..

[3] Matsumoto H (2000). Characterization of a recombinant soluble form of human placental leucine aminopeptidase/oxytocinase expressed in chinese hamster ovary cells. Eur. J. Biochem..

[4] Albiston AL (2001). Evidence that the angiotensin iv (at(4)) receptor is the enzyme insulin-regulated aminopeptidase. J. Biol. Chem..

[5] Saveanu L (2009). Irap identifies an endosomal compartment required for mhc class i cross-presentation. Science.

[6] Pham V (2012). Insulin-regulated aminopeptidase deficiency provides protection against ischemic stroke in mice. J. Neurotrauma..

[7] Loyens E (2011). Deletion of insulin-regulated aminopeptidase in mice decreases susceptibility to pentylenetetrazol-induced generalized seizures. Seizure.

[8] Niwa M (2015). Irap deficiency attenuates diet-induced obesity in mice through increased energy expenditure. Biochem. Biophys. Res. Commun..

[9] Telianidis, J. *et al.* Inhibition of insulin regulated aminopeptidase confers neuroprotection in a conscious model of ischemic stroke. *FASEB J.* (2022).

[10] Vinh A, Widdop RE, Chai SY, Gaspari TA (2008). Angiotensin iv-evoked vasoprotection is conserved in advanced atheroma. Atherosclerosis.

[11] Moeller I (1999). Up regulation of at4 receptor levels in carotid arteries following balloon injury. Regul. Pept..

[12] Uhlen M (2017). A pathology atlas of the human cancer transcriptome. Science.

[13] Rogi T, Tsujimoto M, Nakazato H, Mizutani S, Tomoda Y (1996). Human placental leucine aminopeptidase/oxytocinase. A new member of type ii membrane-spanning zinc metallopeptidase family. J. Biol. Chem..

[14] Nomura M (2002). Differential distribution of placental leucine aminopeptidase/oxytocinase and aminopeptidase a in human trophoblasts of normal placenta and complete hydatidiform mole. Placenta.

[15] FDA-NIH. *Biomarker working group. Best (biomarkers, endpoints, and other tools) resource.*, https://www.ncbi.nlm.nih.gov/books/NBK326791/ (2016). 27010052

[16] Yang H (2011). Angiotensin iv protects against angiotensin ii-induced cardiac injury via at4 receptor. Peptides.

[17] Trocme C (2020). Serum irap, a novel direct biomarker of prediabetes and type 2 diabetes?. Front. Mol. Biosci..

[18] Mostafa TM, El-Gharbawy NM, Werida RH (2021). Circulating irape, irisin, and il-34 in relation to insulin resistance in patients with type 2 diabetes. Clin. Ther..

[19] Guleroglu FY (2022). Fetal pancreas size and maternal serum biomarkers glycated albumin and insulin-regulated aminopeptidase provide no potential for early prediction of gestational diabetes mellitus. Arch. Gynecol. Obstet..

[20] Khaliq OP, Konoshita T, Moodley J, Naicker T (2020). Soluble angiotensin iv receptor levels in preeclampsia: Is there a variation?. J. Matern. Fetal. Neonatal. Med..

[21] Keresztes D (2022). Comparative proteome analysis identified cd44 as a possible serum marker for docetaxel resistance in castration-resistant prostate cancer. J. Cell. Mol. Med..

[22] De Tina A, Juang J, McElrath TF, Baty JD, Palanisamy A (2019). Oxytocin and oxytocinase in the obese and nonobese parturients during induction and augmentation of labor. AJP Rep..

[23] El-Sheikh HM, El-Haggar SM, Elbedewy TA (2019). Comparative study to evaluate the effect of l-carnitine plus glimepiride versus glimepiride alone on insulin resistance in type 2 diabetic patients. Diabetes Metab. Syndr..

[24] Zekry R, Omran GA, El-Gharbawy NM, Werida RH (2023). Comparative study of dapagliflozin versus glimepiride effect on insulin regulated aminopeptidase (irap) and interleukin-34 (il-34) in patient with type 2 diabetes mellitus. Sci. Rep..

[25] Lew RA (2003). Angiotensin at4 ligands are potent, competitive inhibitors of insulin regulated aminopeptidase (irap). J. Neurochem..

