# Supplementary material for: Development of a sandwich ELISA to detect circulating, soluble IRAP as a potential disease biomarker
Source: Sci Rep. 2023 Nov 24;13:17565. doi: 10.1038/s41598-023-44038-1 (PMC10673851; doi:10.1038/s41598-023-44038-1)
Supplement: Supplementary file 1 — Supplementary Information. [file 41598_2023_44038_MOESM1_ESM.pdf]

Supplementary Information

Figure 2a

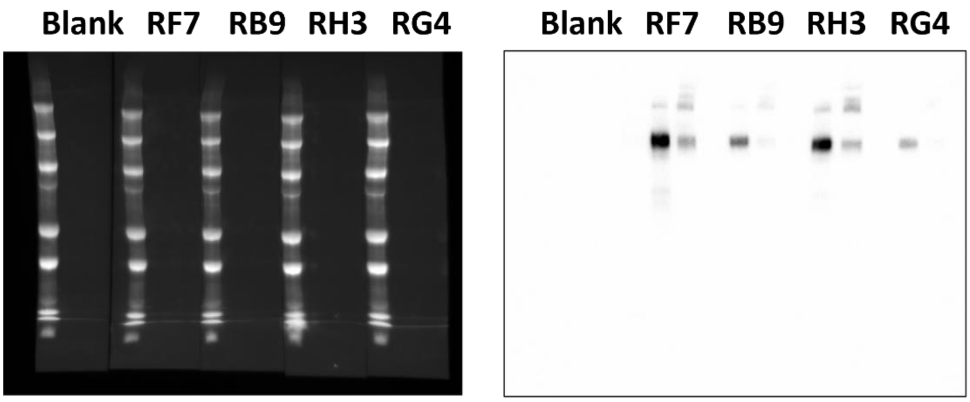

Supplementary Figure 1. *Complete, uncropped Western blots (standards with dark background) presented in Figure 2a. Note the antibodies in the cropped blots in Figure 2a are in a different order.*

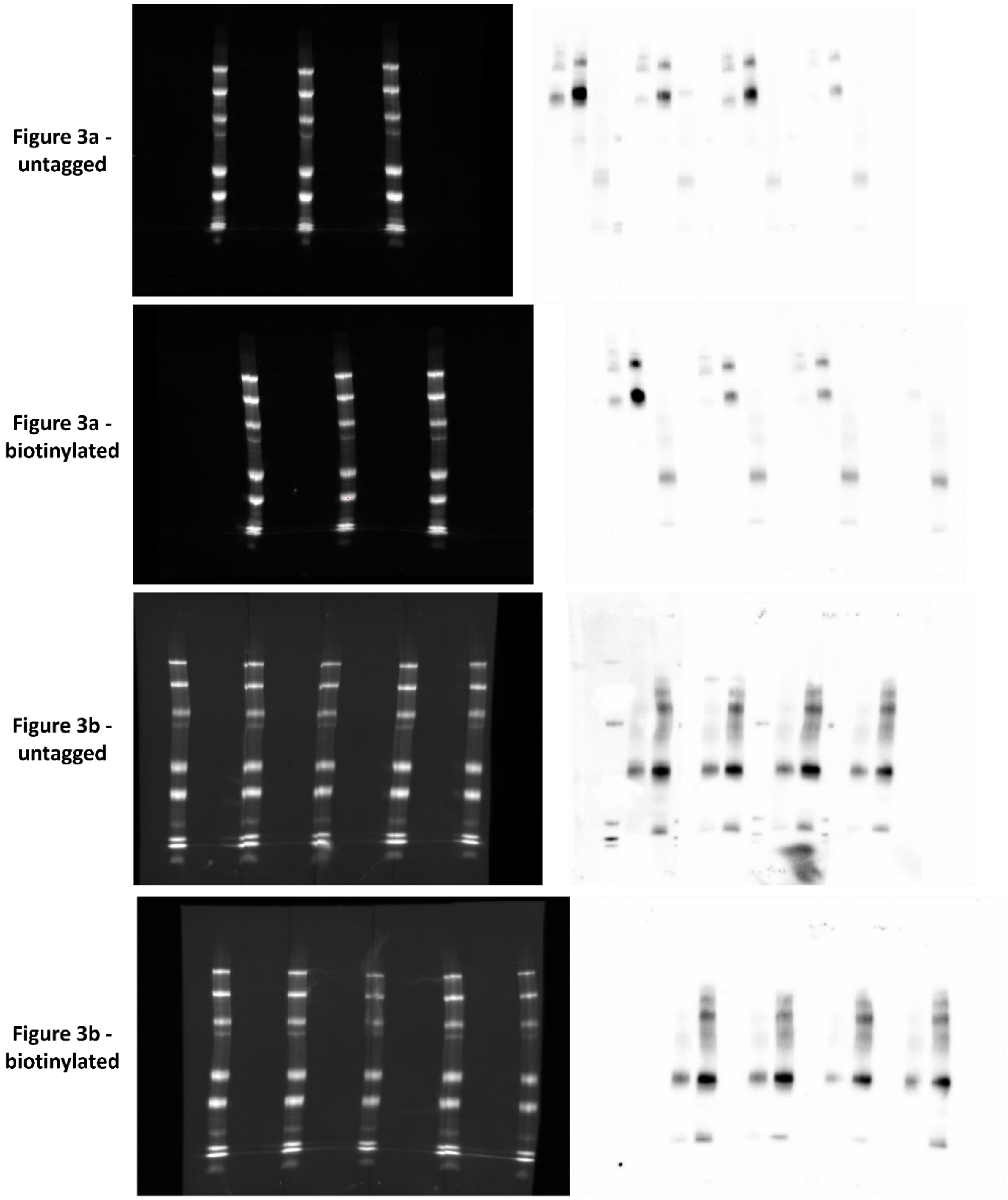

Supplementary Figure 2. *Complete, uncropped Western blots (standards with dark background) presented in Figure 3. All blots are imaged for the same exposure time.*
